# Addressing research integrity challenges: from penalising individual perpetrators to fostering research ecosystem quality care

**DOI:** 10.1186/s40504-019-0093-6

**Published:** 2019-06-10

**Authors:** Hub Zwart, Ruud ter Meulen

**Affiliations:** 1 Erasmus School of Philosophy, Erasmus University Rotterdam, P.O. Box 1738, 3000 DR Rotterdam, The Netherlands; 2 University of Bristol, Office G.04a - Canynge Hall, 39 Whatley Road, Bristol, BS8 2PS UK

**Keywords:** Research integrity, Scientific misconduct, Research performing Organisations, Research ethics

## Abstract

Concern for and interest in research integrity has increased significantly during recent decades, both in academic and in policy discourse. Both in terms of diagnostics and in terms of therapy, the tendency in integrity discourse has been to focus on strategies of individualisation (detecting and punishing individual deviance). Other contributions to the integrity debate, however, focus more explicitly on environmental factors, e.g. on the quality and resilience of research ecosystems, on institutional rather than individual responsibilities, and on the quality of the research culture. One example of this is the *Bonn PRINTEGER Statement*. This editorial to the LSSP thematic series (article collection) entitled *Addressing integrity challenges in research: the institutional dimension* invites authors to contribute to the research integrity debate. Notably, we are interested in submissions addressing issues such as institutional responsibilities, changes in the research climate, duties of research managers and research performing or research funding organisations (RPOs and RFOs) as well as new approaches to integrity education.

## Introduction

Concern for and interest in research integrity has increased significantly during recent decades, both in academic and in policy discourse (Horbach & Halffman 2017). Notably in the public realm, integrity debates are often triggered by spectacular (high visibility) misconduct cases, committed by prominent scientists (or even science celebrities), such as the Schön case (Consoli 2006), the Hwang case (Gottweis & Triendl 2006; Zwart 2008), the Macchiarini case (Vogel 2016) and the Stapel case (Zwart 2017), conveying a common narrative structure, starting with a spectacular ascent, based on fraud, and resulting in a dramatic fall from grace and followed by an avalanche of academic and public comments. Such cases fuel the question how widespread (or even endemic) misconduct practices in contemporary research have become, and how the current wave of integrity challenges in contemporary research can best be addressed. This editorial to the LSSP thematic series (article collection) entitled *Addressing integrity challenges in research: the institutional dimension* invites authors to contribute to the research integrity debate.

This article collection starts from the observation that, both in terms of diagnostics and in terms of therapy, the tendency in integrity discourse has been to focus on the personal ethics and motivations of individuals (individualisation), a tendency which, on the institutional level, concurs with the prevention of damage control (by framing cases of misconduct as individual aberrations). In top-down approaches, individualisation and reputational damage prevention often go hand in hand, we would argue: besides being selectively recruited and closely monitored, individual researchers should know and obey the rules, and should be punished individually if things go wrong. One example of this trend is a publication by Tijdink et al. (2016) which connects research misconduct to the “narcissistic, Machiavellianistic and psychopathic” personality traits of individual researchers. The authors conclude that their main finding (that Machiavellianism is the personality trait that is most strongly associated with research misbehaviour) “may inform those involved in the recruitment of scientific personnel” as well as research managers involved in “integrity monitoring”. In other words, a personality test may increase opportunities for prevention of individual integrity deviance. At the same time, the authors are hesitant when it comes to “translating” their results “directly to the practice”, for example in the context of hiring scientific personnel (p. 10). Rather than being employed as a selection tool, a personality test may increase awareness of these personality traits in researchers and research groups and thus help scientists to gain more insight into and control over their own behaviour during the research process.

Other contributions to the integrity debate, however, focus more explicitly on environmental factors, e.g. on the quality and resilience of research ecosystems, on institutional rather than individual responsibilities, and on the quality of the research culture. An example of this is the paper entitled “Working with Research Integrity—Guidance for Research Performing Organisations”, also known as *The Bonn PRINTEGER Statement* (Forsberg et al. 2018, PRINTEGER 2018). The objective is to advise research managers and research performing organisations and to complement existing instruments by taking into account the daily challenges and organisational contexts of most researchers (the work-floor perspective) and by focusing specifically on institutional responsibilities for strengthening integrity. Not only because, in most disciplines, research is team-work, involving intense collaboration and mutual dependence, but also because many contributors to the debate discern a connection between integrity issues (also in top quality science) and the extent to which the global research arena is becoming increasingly competitive, resulting in wide-spread symptoms such as scientific productivism, the increase of pace and scale, output indicator fetishism and the focus on quantity over quality. In other words, high visibility cases (revolving around exposed science celebrities) seem symptomatic of increasing tensions between performance indicators and quality care.

This was quite obvious in the Hwang case, for instance. Whereas initially comments on Hwang’s scientific “breakthrough” (his claim that he had succeeded on cloning human stem cells) voiced the concern that (in the context of global competition) Asian research “tigers” were out-competing Western science (hampered by ethical constraints), after the misconduct exposure comments in prominent journals such as *Nature* shifted towards a different gear, arguing that ethics and integrity concerns are neither a nuisance nor a constraint, but rather an indispensable aspect of quality care and research governance (Gottweis 2006; Zwart 2008). The question is: do we have our infrastructures for addressing ethics and integrity issues in place? Are we able to address integrity challenges emerging in the global research arena? And who are “we”? Such questions emerge against the backdrop of a broader range of concerns (such as for instance the replication crisis and the concern that trust in and credibility of scientific research is quickly eroding, notably in the post-truth era.

Against this backdrop, integrity has not only become an issue for researchers and research managers, but also for research funding agencies, such as for instance the European Commission. During recent years, numerous calls were published and numerous research projects were or are being funding (with budgets ranging from two to 4 million Euros) to foster research integrity in Europe. This thematic Series was launched by one of these funded projects, namely *Promoting Integrity as an Integral Dimension of Excellence in Research* (PRINTEGER: Swafs 2014-Garri 5; project ID 665926). Building on our results, but also taking into account the results of other projects, we conclude that efforts to foster research integrity should build on two basic recommendations:

1. Fostering research integrity should be a bottom-up process, informed by practice, by integrity work in every-day research settings
2. First and foremost, research integrity should be strengthened, not via individualisation (i.e. surveillance, detection, exposure and punishment of individual deviance) but via institutionalisation (i.e. promoting care and concern for research ecosystem quality)

In response to how the international research climate is changing (the rise of big science, the increase of scale and pace of research, the attention given to quantifiable performance indicators for funding or assessing research, etc.) and in order to address the integrity challenges entailed in them, research institutes (notably universities) should strengthen research integrity by fostering a culture of deliberation, by facilitating open dialogue and by creating a safe environment for identifying and discussing integrity issues emerging in daily practice. Rather than applying norms and guidelines in a top-down manner, or focussing on reputational damage repair, research institutes should provide the conditions which allow collective responsibility to flourish.

Although codes and guidelines (such as the *European Code of Conduct for Research Integrity*, ALLEA 2017) are important, codes require a resilient integrity culture to be effective. Codes may provide guidance to the extent that they are informed by accumulated experiences. And they may bring to our attention questionable practices which have become routines but should actually be reconsidered. Indeed, they allow us to articulate what is often taken for granted, so that we can reassess established practice. In real practice, however, where dilemmas can be quite unique, such codes may often prove too general. Therefore, they need a context, a supportive research environment to work. Codes have to be practiced and internalised and require a culture of deliberation to have an impact. Therefore, in the current integrity debate, besides codes, we need to *care* for our codes. Integrity care focusses on personal relationships, attentiveness, responsiveness, dialogue, competence and context (Tronto 2005). Rather than operating as solitary individuals, researchers tend to be highly dependent on one another. Although the current focus on codes and guidelines is understandable and laudable in itself, they often function as straightjackets if insufficient attention is given to institutional responsibilities, first and foremost for fostering conditions for quality care. While on the institutional level strategies of individualisation are often used to prevent reputational damage, we advocate the endorsement of an attitude of openness, transparency and deliberation, resulting in sharing experiences and mutual organisational learning. Likewise, funding agencies could focus less on quantifiable performance indicators and more on *good science*, which may be time-consuming, also because sensitivity to societal concerns will become an inherent dimension of the research’s methodology.

This shift of focus from individual deviance to institutional quality care should be the starting point, not only for developing integrity policies, but also for designing educational tools for future researchers. Whereas current integrity teaching (e.g. the interactive integrity module *The Lab*, developed by the NIH Office of Research Integrity) often focus on individual dilemmas and decisions, next generation educational tools should bring the institutional context and responsibilities more explicitly into view, so that the primary question no longer is: what should be my decision as an individual researcher facing a particular dilemma, but rather: how could this dilemma emerge in the first place? Rather than solving integrity puzzles, the focus should be on fostering a research environment of deliberation and shared responsibilities. Thus, a wider set of instruments becomes available for research managers to create a research climate where integrity challenges can be successfully met and where individual integrity dilemmas can be placed in a broader context, as symptoms of more general developments. The focus of attention will shift to integrity team work: from how to prevent individual fraud to how to address potentially disruptive trends (e.g. increase of competition, focus on quantifiable performance indicators, etc.) and the perverse incentives to which they may give rise (indicator fetishism, output steering, h-factor obsession, etc.).s

Via this editorial, we want to invite participants in the academic and policy debate to share their views on how to foster research integrity, paying special attention to issues such as institutional responsibility, changes in the research climate, duties of research managers and research performing or research funding organisations (RPOs and RFOs) as well as new approaches to integrity education.

## Abbreviations

ALLEA: All European Academies
NIH: National Institutes of Health
PRINTEGER: Promoting Integrity as an Integral Dimension of Excellence in Research
RFO: Research funding organisation
RPO: Research Performing Organisation
SWAFS: Science with an for Society

## Authors’ information

Hub Zwart (1960) studied philosophy and psychology at Radboud University Nijmegen, worked as a research associate at the Centre for Bioethics in Maastricht (1988–1992) and defended his thesis in 1993. In 2000 he became full Professor of Philosophy at the *Faculty of Science* RU Nijmegen and in 2018 he was appointed as Dean of *Erasmus School of Philosophy* (Erasmus University Rotterdam). He published 15 books (4 in English) and > 100 academic papers. He is editor-in-chief of the *Library for Ethics and Applied Philosophy* (Springer) and of the journal *Life Sciences, Society and Policy* (Springer). In his research he develops a continental philosophical assessment of contemporary technoscience. Special attention is given to genres of the imagination (novels, plays, poetry) in research and education.

Ruud ter Meulen is particularly interested in justice in health care, the ethics of research, care of older people, and evidence-based medicine. I have directed several international research projects, including large-scale projects funded by the European Commission, and have published extensively in the field of bioethics. I am the Director of the Centre for Ethics in Medicine. I was educated in psychology (Catholic University of Nijmegen), an area in which I worked for some years, before moving into the field of bioethics. I was previously the Director of the Institute for Bioethics and Professor for Philosophy and Ethics at the University of Maastricht (1995–2005).
